# Drug susceptibility profiles of Mycobacterium abscessus isolated in the state of São Paulo, 2008–2024

**DOI:** 10.1099/jmm.0.002005

**Published:** 2025-04-15

**Authors:** Carolina Salgado Pedace, Robert D. Arbeit, Fernanda Cristina dos Santos Simeão, Juliana Failde Gallo, Andréia Rodrigues de Souza, Erica Chimara

**Affiliations:** 1 Bacteriology Center, Adolfo Lutz Institute, São Paulo/SP, Brazil; 2 Division of Infectious Diseases, Tufts Medical Center, Boston, MA, USA

**Keywords:** minimal inhibitory concentration, non-tuberculous mycobacteria, resistance

## Abstract

**Introduction.** Infections caused by *Mycobacterium abscessus*, an environmentally prevalent, rapidly growing mycobacteria, are increasingly frequent in developed countries.

**Objective.** To analyse the drug susceptibility profiles of *M. abscessus* isolated in the state of São Paulo from 2008 to 2024.

**Methods.** Of the 2,402 *M*. *abscessus* isolates identified during those 17 years, 558 (23.2%) met the American Thoracic Society’s microbiologic and clinical criteria for drug susceptibility testing (DST), which was performed for five agents – clarithromycin, amikacin, cefoxitin, ciprofloxacin, and doxycycline.

**Results.** Clarithromycin showed a dramatic increase in resistance phenotype from ≤10% in the early period to 73–90% over the last 8 years. Over half those isolates demonstrated inducible resistance. Resistance to amikacin was found in fewer than 5% of isolates from 2016 to 2021. In 2022, that result increased to 13%, but for 2023 and 2024, it had fallen back to 2%. Over the past decade, cefoxitin DST has reported the majority of isolates as intermediate, a problematic result in *M*. *abscessus* group (MAG) infections, which typically require long-term treatment for successful outcomes. Since 2018, the annual susceptibility rate has been ≤18%, and in five of the 7 years, ≤7%. Ciprofloxacin was typically assessed as susceptible from 2009 to 2011, then decreased sharply to ≤20% over the next several years, and since 2018, the rate has been less than 5%. Through the entire study, doxycycline resistance has remained consistently high; in the years since 2018, ≤6% of isolates have been susceptible.

**Conclusion.** This study demonstrates wide variation among MAG clinical isolates in the frequency of susceptibility, both across different agents and within individual agents over time. These results emphasize the importance of performing high-quality DST on MAG clinical isolates and suggest the need to consider revising the standard panel of drugs tested.

## Introduction

Non-tuberculous mycobacteria (NTM) are widely present in the environment, including soil, groundwater and biofilms associated with municipal water sources [1,3]. In recent years, the incidence of both symptomatic and asymptomatic clinical infections caused by these organisms has increased [4]. The lung is the most common site of infection, and NTM pulmonary infections have proven particularly difficult to treat in patients with cystic fibrosis and chronic lung disease [5,6]. Focal infections of other tissues (e.g. lymph nodes, skin and soft tissue), as well as disseminated disease, can occur in both immunocompetent and immunocompromised patients [7,8].

Infections due to *Mycobacterium abscessus*, a rapidly growing mycobacteria (RGM), have increased appreciably in developed countries [9,10]. There are three subspecies of *M. abscessus* – subsp. *abscessus*, *bolletii*, and *massiliense* – collectively referred to as the *M. abscessus* group (MAG). Over the past three decades, outbreaks of MAG infection have been reported in North and Latin America, as well as East Asia [11,18]. Brazil has experienced major outbreaks in 15 states [19,23], including clusters associated with video-assisted surgeries using equipment disinfected with 2% glutaraldehyde solution, to which MAG are intrinsically resistant [21].

The current consensus is that the diagnosis of NTM pulmonary infection requires both clinical and microbiological assessments to rule out other diagnoses, particularly tuberculosis and malignancy [24]. Specific bacteriological criteria for NTM pulmonary infection include positive cultures from at least two sputum samples, at least one bronchial lavage, or a lung biopsy showing granulomas and/or acid-fast bacilli (AFB), plus a positive culture from the biopsy or from at least one sputum or bronchial washing [24]. For sterile sites, including blood, biopsies and body fluids, a single positive culture is sufficient [4,25, 26].

From the time of diagnosis, infections due to MAG are particularly challenging to treat for two related reasons. Among the potentially pathogenic RGM, clinical MAG isolates have the highest rates of antimicrobial resistance [5,11, 27]. Notably, MAG are routinely resistant to agents commonly used to treat tuberculosis, including isoniazid, rifampicin, ethambutol and pyrazinamide [7,12]. Furthermore, during treatment, infecting strains that were previously sensitive to the prescribed agents can acquire resistance mutations [4,28,30]. Consequently, successful treatment of MAG infections typically requires an extended course of 12–18 months.

In response to these issues, the Clinical and Laboratory Institute (CLSI) recommends that, at the time of diagnosis, clinical isolates of MAG have prompt and accurate drug susceptibility testing (DST) to determine the minimum inhibitory concentrations (MICs) and identify suitable agents for treatment [26]. Furthermore, during the course of treatment, follow-up cultures should be periodically obtained, even from patients without clinical changes, and any recovered isolates should also undergo full DST. Even after an apparently successful course of treatment, recurrent disease is, unfortunately, all too common and requires comprehensive repeat cultures and DST of any isolates [31].

Only agents representing two drug classes – aminoglycosides and macrolides – have proven clinically effective in the treatment of MAG infections. The aminoglycosides, amikacin and tobramycin, require parenteral administration [24,32, 33]. While the macrolides, clarithromycin and azithromycin, can be given orally [32], their use is increasingly compromised by the rising prevalence of inducible resistance [28], which (as detailed below) requires specialized DST procedures to be detected. Beta-lactams are typically inactivated by a broad-spectrum beta-lactamase (*bla*_Mab_) common among MAG [25], although some (e.g. cefoxitin, imipenem and meropenem) have moderate *in vitro* activity.

The Adolfo Lutz Institute is a reference laboratory for the identification and DST of mycobacterial isolates from patients in the state of São Paulo, the most populous region in Brazil. The aim of the present study was to analyse the drug susceptibility profiles of clinical isolates of MAG cultured in the state of São Paulo from 2008 to 2024.

## Methods

**Fig. 1.**
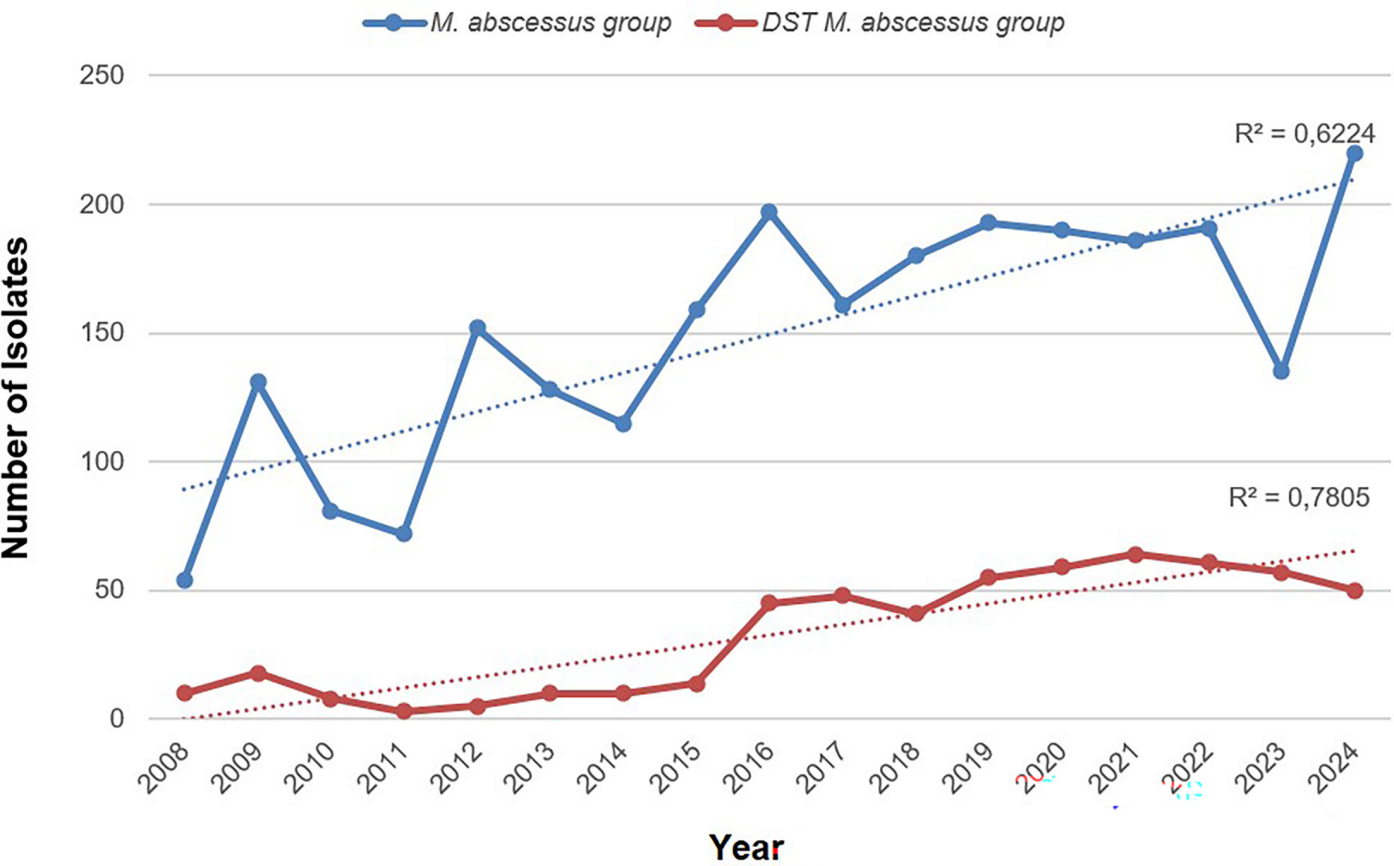
Total number of isolates identified as MAG and the number for which DST was performed from 2008 to 2024. The increase in DST after 2015 reflects the initiation of selecting isolates for testing based on bacteriological criteria as recommended by CLSI (see text for details). Adolfo Lutz Institute, São Paulo, Brazil.

The present study was a retrospective analysis of DST results from 2008 to 2024 for clinical isolates of MAG. From 2008 through 2015, DST of MAG was primarily performed at the request of the clinician, resulting in limited and variable numbers tested, ranging from 3 to 18 isolates annually (Fig. 1). In 2016, bacteriological criteria for DST were introduced in the laboratory [4,34], and the number tested rose sharply to 45 isolates. It has continued to increase, ranging from 50 to 64 isolates over the last 5 years of this study (2020 to 2024).

Species identification was performed using molecular techniques, including the PRA-h*sp*65 method [35] (2008–2019), Speed Oligo test® (Vircell, Granada, Spain) [31] (2016–2021) and Genotype NTM-DR® (Hain LifeScience, Nehren, Germany) [36] (2021–2024). The drugs tested were clarithromycin (CLA), amikacin (AMK), cefoxitin (FOX), ciprofloxacin (CIP) and doxycycline (DXT), as currently recommended by CLSI [26].

MICs were determined by broth microdilution, as recommended by CLSI [26] and modified by Carvalho *et al*. to include the following. After 3 days of incubation at 35±2 °C, a visual reading of the positive controls was performed to confirm adequate growth. Subsequently, 30 µl of 0.1% resazurin solution was added to all inoculated wells, the plates were reincubated and readings were taken after 24 hours. The colour change from blue (oxidized state) to pink (reduced) indicates bacterial growth and has been confirmed to increase reading reliability [36,39].

To standardize the categorical results across the 17 year study period, all MICs were classified as susceptible, intermediate or resistant based on the current CLSI breakpoints [40] (Table 1).

**Table 1.** Breakpoints for antimicrobial agents recommended for DST of MAG isolates

Antimicrobial agent	MIC (µg/ml)			Comments*
	S	I	R	
Clarithromycin	≤ 2	4	≥ 8	See CLSI document M24^33^ for guidance regarding incubation period, molecular testing and interpretation of clarithromycin results for rapid-growing mycobacteria. Clarithromycin is the class representative for the newer macrolides (i.e. azithromycin and roxithromycin).
Amikacin (IV)	≤ 16	32	≥ 64	–
Cefoxitin	≤ 16	32–64	≥ 128	–
Ciprofloxacin	≤ 1	2	≥ 4	Ciprofloxacin and Levofloxacin are interchangeable. Both are less active *in vitro* than the newer 8-methoxy fluoroquinolones.
Doxycycline	≤ 1	2–4	≥ 8	-–

*The comments shown are excerpts from CLSI [41].

I, intermediate resistance; IV, intravenously. R, resistance; S, susceptible;

### Preparation and configuration of DST plates

DST was performed in the IAL laboratory using sterile plates with 96 U-shaped microwells arranged in 8 rows and 12 columns.

Stock solutions of each drug (Sigma-Aldrich, Merck, Darmstadt, Alemanha) at a concentration of 1% (1 μg/100 µl) were prepared in sterile distilled water, and 100 µl aliquots were stored at –20 °C ± 1^33^. When the plates were prepared for testing, an aliquot of the stock solution for each drug was diluted in cation-adjusted Mueller Hinton Broth (caMHB) (BD, New Jersey, EUA) to twofold the highest concentration needed. MHC (100 µl) was added to all wells, then 100 µl of the freshly diluted antibiotic was dispensed into the first well of the row, followed by serial twofold dilutions made to the lowest concentration required, using no more than eight wells. In all rows, the remaining wells included three growth controls without antibiotics and a minimum of one sterility control with no organisms. The plates were prepared in batches to permit testing of up to 40 isolates. Each plate was promptly packaged, stored at –20 °C ± 1, and typically used well before the 3-months expiration date.

### Mycobacterial inoculum preparation

The stored clinical MAG isolates were first subcultured on solid Lowenstein-Jensen (LJ) medium at 35 ± 2 °C for 3 days, then in MHC for another 3 days. The isolate inoculum for DST was prepared by adding two or three drops of that culture to tubes containing 3 ml of fresh MHC and monitoring optical density at 625 nm using a spectrophotometer (Celm, Brazil). When absorbance reached 0.08 to 0.10 (representing McFarland scale 0.5), the suspension was diluted 1 : 100, and 100 µl aliquots were added to each drug-containing well and the growth control wells. The plates were covered, placed in plastic bags to prevent evaporation of the medium, stored in a sealed box and incubated at 35 ±2 °C.

### Plate incubation and reading

From 2008 to 2015, each plate contained all five antibiotics, was inoculated with a single organism, and on Day 3 of incubation, bacterial growth was assessed using the resazurin procedure detailed above and the MIC was determined. From 2016 to the present, that approach continued for AMK, FOX, CIP and DXT. However, handling CLA had to be modified to accommodate the procedures required for differentiating constitutive and inducible resistant.

For the CLA plates, each isolate was inoculated into two rows per plate. On Day 3, the resazurin procedure was performed for one row, and organisms that showed growth at a CLA concentration ≥8 µg ml^−1^ were designated with acquired resistance. The plate was then reincubated, and the replicate row for each organism was read on Day 14. Inducible resistance to CLA was defined as a reading indicating limited growth on Day 3 that subsequently proved to represent a false susceptibility result when the reading on Day 14 demonstrated robust growth, indicating resistance. That pattern is consistent with the induction of methylase in an MAG isolate growing in the presence of CLA [26,35,41].

### Quality controls

The following quality control procedures were performed for each batch of plates produced and for each DST performed, as recommended by CLSI [40].

At the time each batch of test plates was prepared, the quality of the media and the antibiotic dilutions was assessed using the control organisms *Staphylococcus aureus* ATCC^®^ 29213, *Pseudomonas aeruginosa* ATCC^®^ 27853 and *Enterococcus faecalis* ATCC^®^ 29212. Each organism was tested in triplicate wells.

At the time plates were used for DST, the growth control organism *Mycobacterium peregrinum* ATCC^®^ 700686 was inoculated into triplicate wells of one plate with the four antibiotics and one plate with CLA. Concurrently, the culture quality of the clinical isolate inoculum was assessed by inoculating two drops onto a blood agar plate. If contamination with routine bacteria or another species of NTM was detected, the associated DST plates were discarded, a fresh inoculum prepared and confirmed, and testing was repeated on an additional set of plates.

## Results

Over the 17 years of this study, 28,617 isolates of NTM were received at the Adolfo Lutz Institute, including 2,402 (8.4%) identified as MAG, of which DST was performed on 558 (23.2%). Of those clinical isolates, the substantial majority (74.6%) were from pulmonary specimens; no other sample source represented more than 6% of the isolates (Table 2).

**Table 2.** Distribution of 558 clinical MAG isolates by site of isolation

Source	No. of isolates (%)	Details
Pulmonary	416 (74.6%)	371 sputum, 39 bronchial washing, 3 tracheal aspirate, 2 lung fragment, 1 thorax secretion
Wounds	33 (5.9%)	7 surgical, 4 gluteal abscesses, 2 thigh abscesses, 1 knee abscess, 1 vertebral abscess, 1 hip abscess, 1 abdomen abscess, 16 unspecified wounds
Breast secretions	26 (4.7%)	–
Tissue biopsies	22 (3.9%)	7 skin, 5 bone, 1 bladder, 1 endocervical, 1 muscle, 7 unspecified
Body fluids	18 (3.2%)	5 peritoneal, 6 urine, 2 ocular, 2 gastric, 2 pleural, 1 cerebrospinal fluid
Blood	11 (2.0%)	–
Unreported sites	32 (5.7%)	–

**Fig. 2.**
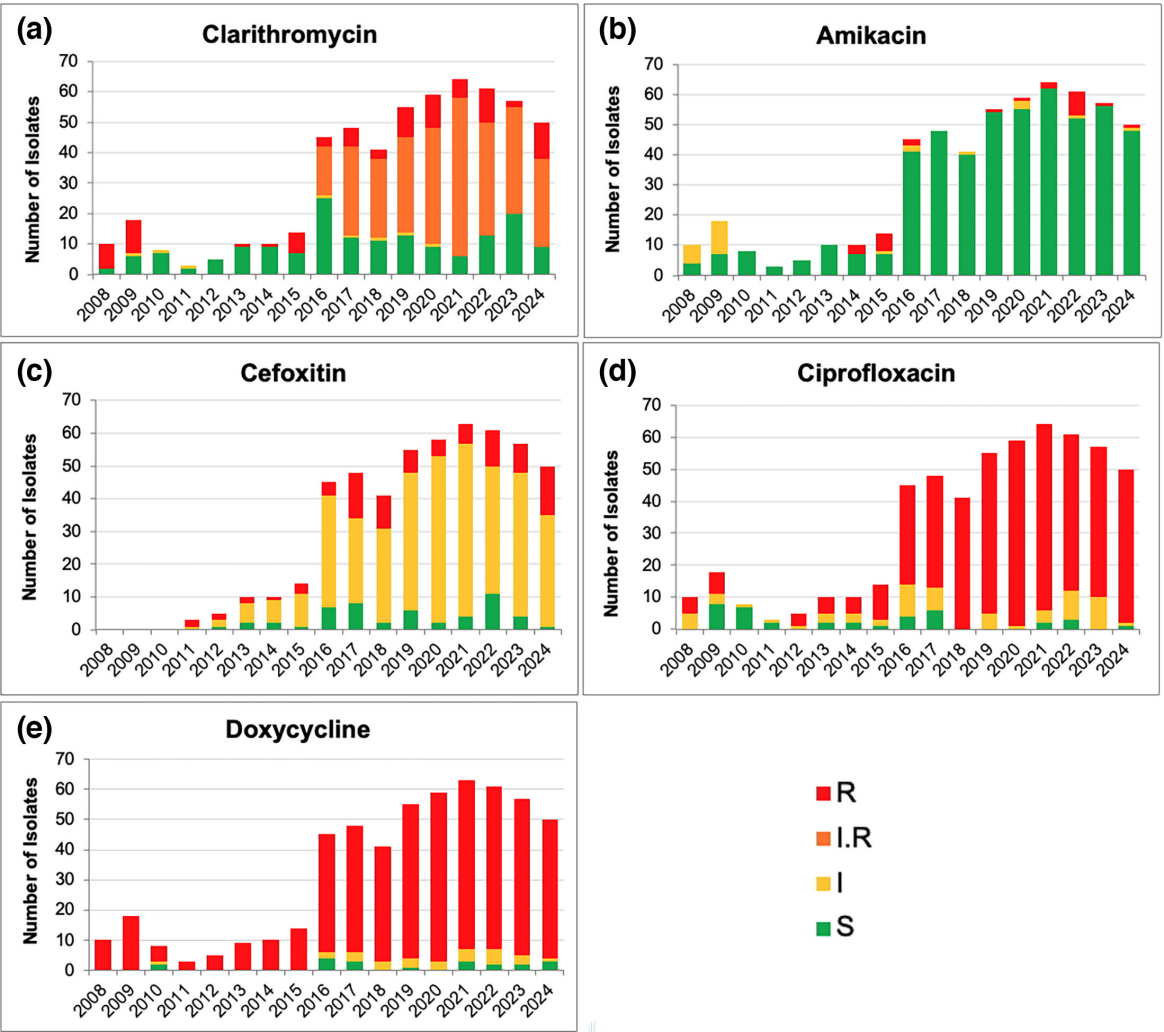
Susceptibility profiles for five drugs in MAG isolates from 2008 to 2024. The graphs show the profiles of clarithromycin (a), amikacin (**b**), cefoxitin (**c**), ciprofloxacin (**d**) and doxycycline (**e**). DST result labels: I, intermediate; I.R, induced resistance; R, resistant; S, susceptible. Note: For clarithromycin, beginning in 2016, resistance (**r**) is based on readings taken on Day 3 of incubation; IR is based on readings from Days 4 through 14 of incubation. Institute Adolfo Lutz, São Paulo, Brazil.

The limited annual data prior to 2016 have inherently greater random variation in the distribution of DST results. Nevertheless, over the entire study period, each of the five antimicrobial agents tested shows a different pattern of change in the nature and frequency of susceptibility phenotypes (Fig. 2).

Clarithromycin (Fig. 2a) shows the most complex change, with overall resistance ≤10% from 2010 to 2014, increasing to 50% in 2015 and ranging from 73% to 91% over the last 8 years of the study (2017–2024). During this period, DST testing for CLA included an extended 14-day observation, which demonstrated inducible resistance as the dominant phenotype, representing 60–81% of all isolates.

For amikacin (Fig. 2b), >90% of isolates have typically been susceptible. The variation observed in some early years (2008–2009 and 2014–2015) likely reflects the limited number of isolates. From 2016 to 2020, <5% of isolates were consistently resistant. That increased to 13% in 2022. But in 2023 and 2024, the last 2 years of this analysis, resistance was only ≤2%.

Since 2013, cefoxitin (Fig. 2c) DST has indicated the majority of isolates as intermediate, and since 2018, only 3–18% as susceptible. The clinical utility of cefoxitin for intermediate susceptible MAG isolates has not been established [24,42].

Ciprofloxacin (Fig. 2d) was assessed as typically susceptible (44–87%) in the period of 2009 to 2011, decreasing sharply to ≤20% over the next several years (2012–2017) and since 2018, has been <6%.

Resistance to doxycycline (Fig. 2e) has been demonstrated for ≥86% of isolates every year except 2010. Since 2018, ≤6% of isolates have been susceptible.

## Discussion

MAG is recognized as an emerging pathogen in Brazil and globally [43]. MAG, like other NTM, is an environmental organism, and the most common site of infection is the lungs; patients with chronic lung disease and cystic fibrosis are at particular risk [44]. In the state of São Paulo, MAG was identified as the most frequently isolated NTM as early as 2014, and among all mycobacteria, it remains second only to *M. tuberculosis* [45]. Notably, in Brazil in 2020, during the COVID-19 pandemic, the frequency of sputum samples positive for *M. tuberculosis* decreased by 34.6% [46], while in the state of São Paulo, the frequency of MAG isolates remained stable. This observation is consistent with the perspective that MAG pulmonary infection is acquired primarily from the environment and rarely transmitted person-to-person, although outbreaks among cystic fibrosis patients have been described [43].

This report presents 17 years of DST results for MAG isolates performed by microbroth dilution assay for five agents formally recommended by CLSI in 2018, each representing a different class of antimicrobials [40]. During the initial period (2008–2015), our laboratory performed DST for MAG isolates primarily when requested by the clinician. This practice resulted in the assay of an average of 7.3 (range 3–18) isolates per year and likely contributed to the higher frequency of resistance observed across all five antimicrobials during that period. Beginning in 2016, we have applied bacteriological criteria to select isolates [4,40], resulting in an average of 53 (range 41–64) isolates per year being tested. This sevenfold increase has provided more clinically relevant susceptibility profiles for the different antimicrobials and offers the potential for more timely detection of emerging resistance to individual agents.

During the most recent 9 years, DST of CLA included a 14-day extended incubation, which consistently demonstrated inducible resistance in over 50% of the MAG isolates and constitutive resistance in 4–19%. Inducible resistance is mediated by an erythromycin ribosomal methylase encoded by *erm*(14), present in MAG subspecies *abscessus* and *bolletii*. The subspecies *massiliense* carries a truncated, inactive form of the gene [47,48]. During treatment, all MAG isolates can develop acquired resistance mediated by mutations in the 23S rRNA gene [49,51].

CLA is essentially the only agent for which *in vitro* susceptibility has been convincingly associated with sustained sputum clearance and a successful clinical outcome [49,51]. MAG infection can resolve in 84% of isolates sensitive to CLA, but that rate drops to 23% for strains with constitutive or inducible resistance [24,52,57]. In our laboratory, the frequency of CLA-susceptible MAG isolates ranged from 24% to 56% from 2016 to 2019, but in four of the last 5 years, it has been 9% to 21%. In the same period, global rates of susceptible isolates varied widely across diverse locations [58,64], ranging from greater than 60% in Japan [63] and the United Kingdom [64] to less than 20% – similar to ours – in North Korea [58] as early as 2014 and more recently in the United States [59], South Korea [60] and Iran [61]. In MAG, most of the change represents the inducible resistance phenotype [28,62]. The widespread and increasing use of macrolides as monotherapy in general medical care is considered a key contributor to the selection of resistance in MAG and other NTM isolates [65]. The source of the geographic variability remains unclear.

AMK is consistently the drug with the highest susceptibility rate for MAG isolates [56,60, 62, 65]. From 2016 to 2024, we observed annual rates of 91–100% in all but 1 year. In 2022, the susceptibility rate dropped to 85%, raising concern. However, in the past 2 years, over 96% of MAG isolates have tested as susceptible. For isolates sensitive to both agents, the combination of parenteral AMK and an oral macrolide has been shown to be the regimen that is most reliably considered to have the best chance of preventing the emergence of mutational resistance to both drugs [66]. However, sustained administration of parenteral amikacin is complicated by both the requirement for intravenous access and relatively higher rates of drug-related adverse events. These have prompted increased interest in inhaled amikacin, but it remains unclear if this route of administration has similar therapeutic efficacy in MAG pulmonary infections [66,67].

FOX is often reported to have high rates of resistance among MAG isolates [68]. However, our observations for the drug have been more complex. Over the period from 2016 to 2024, the annual rate of susceptible isolates has varied between 2% and 18%, and that of resistant isolates between 8.6% and 30%, but neither has shown a clear secular trend. Over the same period, the majority (54–88%) of MAG isolates have consistently tested as intermediate susceptible, representing a relatively narrow range of MICs (32–64 µg ml^−1^). Cefoxitin is typically reported as having a high rate of susceptible MAG isolates, and there have been only a few publications with results similar to ours. Two articles from China – Lee et al. (2017) [62] and He et al. (2022) [42] – describe rates of approximately 84% intermediate resistance to FOX [42,62]. There is no clear explanation for the apparent wide variability. Regardless, during the extended course of treatment required for MAG infections, isolates with intermediate susceptibility are particularly likely to become fully resistant, making FOX a problematic antimicrobial for use in MAG infections.

Both CIP and DOX have high resistance rates among MAG across multiple studies [60,62]. Since 2018, the frequency of susceptible isolates has been consistently ≤5% for both agents. The high frequency of resistance likely reflects the decades of using fluoroquinolones and tetracyclines for the treatment of numerous infections associated with multiple different pathogens [69]. Regardless, in the context of serious MAG infection among patients in our area, neither class of antimicrobials is of practical use.

Since 2020, there have been several reviews and guidelines addressing the treatment of NTM infections in general and MAG infections in particular [24,66, 70]. Unfortunately, the most consistent perspective is summarized in a single sentence in the guidelines for MAG pulmonary disease from IDSA and ERS: ‘the optimal drugs, regimens, and duration of therapy are not known’ [24]. This conclusion reflects the lack of rigorous clinical studies that support a clear correlation between DST *in vitro* and clinical outcomes in patients. That deficit reflects the intersection of several complicating factors. While MAG is a relatively frequent pathogen among NTM, the absolute number of cases is still modest. Furthermore, the need to administer multiple antibiotics concurrently for a year or more is frequently associated with the need to change the regimen due to on-treatment drug-related toxicity and/or emergence of resistance.

Nevertheless, in 2018, CLSI published recommendations regarding both diagnosis and treatment based on collective experience [34,39]. Performing DST is essential not only prior to initiating treatment but also on isolates recovered during treatment from regularly collected sputa. In 2020, IDSA, ERS and other societies published clinical guidance on drug selection and duration of treatment of NTM pulmonary infection (Table 3) [24]. Specifically, at least two, and in some contexts three or four, antibiotics of different classes to which recent isolates are susceptible should be administered for 12 months. The primary factor distinguishing the panels of drugs proposed for consideration is whether the target organism is CLA-susceptible. The extended treatment required is considered in two phases. During the initial phase, typically up to 3 months, the recommended regimen includes one or two parenteral agents and two to three oral agents. The continuation phase that follows to complete treatment includes two or three oral drugs plus inhaled amikacin [24].

**Table 3.** Treatment regimens for *Mycobacterium abscessus* by macrolide susceptibility (mutational and inducible resistance)

Macrolide drug susceptibility*	No. of preferred drugs recommended†			
	Initial phase‡			Continuation phase§
	Total	Parenteral¶	Oral**	Oral *plus* inhaled ††
**Susceptible**	≥3	1 or 2	2	2 or 3
**Resistant‡‡**	≥4	2 or 3	2 or 3	2 or 3

Adapted from ATS, 2020 [19,24].

*All MAG should have drug susceptibility testing (DST) using the CLSI 14-day incubation protocol [25].

†All drugs should be dosed daily, except amikacin, which may be administered three times per week.

‡The initial phase refers to the period when treatment includes parenteral agents; the duration is determined by physician assessment of clinical response, sputum culture and chest x-ray.

§The continuation phase refers to the subsequent period of therapy, which, in addition to the recommended oral agents, may include inhaled amikacin.

¶Parenteral: Amikacin, Imipenem (or Cefoxitin), Tigecycline. The effectiveness of cefoxitin against MAG of intermediate susceptibility is uncertain. Optimal dosing of tigecycline has not been established for NTM.

**Oral: Clarithromycin (or azithromycin), Clofazimine, Linezolid. Note: Based on published data, clarithromycin is clinically active against susceptible MAG and should be used whenever possible.

††Oral plus inhaled: includes inhaled amikacin.

‡‡For infections with clarithromycin-resistant isolates, frequent on-treatment cultures are recommended to detect the emergence of additional MAG resistance or super-infection with *M. avium* complex or other resistant NTM.

There are challenges in applying even these relatively recent guidelines. Of the five agents recommended for MAG by CLSI in 2018 [26], our recent DST results appear to eliminate three – FOX, DXT and CIP (and, presumably, moxifloxacin). The alternatives more recently proposed for consideration include linezolid and bedaquiline [24]. Omadacycline, which was approved in late 2018, has attracted recent interest because it is typically effective in the presence of resistance to generic tetracyclines [71,73], is available for both oral and intravenous administration and is well-tolerated. A recent multicentre retrospective report [71] detailed 117 patients with MAG infection whose treatment included omadacycline for a median duration of 8 months (IQR, 4–15 months), with 48.7% of patients still on treatment at the time of data collection. Adverse events attributed to omadacycline were reported in 30% of patients, with 20% treatment discontinuation. The most common event (21% of patients) was nausea, with or without vomiting, with discontinuation in half of these cases. Only three patients had events involving hepatic or haematologic findings, none of which were life-threatening. Outcome findings are difficult to assess for a non-protocol dataset involving an infection requiring multiple agents. However, the authors considered omadacycline as having ‘potentially promising effectiveness’, given that among 70 isolates with comprehensive macrolide DST, only 15 (21%) were susceptible, with the rest showing inducible resistance.

However, changing the standard panel for DST represents a substantial effort requiring consensus and collaboration among multiple sets of professionals. Implementation would require carefully executed laboratory studies, as well as at least preliminary clinical trials or consistent observation reports. Even then, successful application of the new laboratory results would require thoughtful discussions with clinical colleagues treating patients with MAG and other mycobacterial infections. An additional issue is that several candidate agents suggested – linezolid, bedaquiline and clofazimine – are used in the current regimens for MDR-TB. An increase in the prevalence of resistance in this context could have both clinical and public health impact. Nevertheless, formally validating DST procedures for agents potentially more relevant to the treatment of MAG infection is essential for increasing the rates of successful treatment in current patients and is a prerequisite for developing clinical data addressing whether *in vitro* antimicrobial susceptibility of MAG is meaningfully associated with *in vivo* efficacy [4,74].

In summary, our analysis of 17 years of DST results for five agents recommended for MAG infections demonstrates two sobering conclusions. First, there has been a sustained increase in resistance to three agents such that they appear no longer relevant. Second, the progressive decrease in the frequency of CLA-susceptible MAG isolates likely heralds a decrease in successful clinical outcomes. These data reinforce the critical need for the microbiology laboratory to routinely identify the species of NTM clinical isolates, perform DST of the appropriate agents and ensure that the treating clinician is aware of and understands the therapeutic implications of the laboratory results.

## Supplementary material

10.1099/jmm.0.002005 Uncited Table S1.

## Abbreviations

AFB: acid-fast bacilli
AMK: Amikacin
ATCC: American Type Culture Collection
caMHB: cation-adjusted Mueller Hinton Broth
CIP: Ciprofloxacin
CLA: Clarithromycin
CLSI: Clinical and Laboratory Institute
DST: drug susceptibility testing
DXT: Doxycycline
ERS: European Respiratory Society
FOX: Cefoxitin
IDSA: Infectious Diseases Society of America
LJ: Lowenstein-Jensen
MAG: *M. abscessus* group
MHC: Mueller Hinton cation
MIC: Minimum inhibitory concentration
MIC: minimum inhibitory concentration
NTM: non-tuberculous mycobacteria
RGM: rapidly growing mycobacteria
