# Supplementary material for: Drug susceptibility profiles of Mycobacterium abscessus isolated in the state of São Paulo, 2008–2024
Source: J Med Microbiol. 2025 Apr 15;74(4):002005. doi: 10.1099/jmm.0.002005 (PMC12282310; doi:10.1099/jmm.0.002005)
Supplement: Uncited Table S1. [file jmm-74-02005-s001.pdf]

Suppl. Table 1. Number and Percent of Isolates in Each Susceptibility Category by Year and Antimicrobial

	Year	Susceptible		Intermediate		Inducible Resistant		Resistant		Total
		N	Percent	N	Percent	N	Percent	N	Percent	N
**Clarithromycin**	**2008**	2	20%	0	0%	[a]		8	80%	10
	**2009**	6	33%	1	6%			11	61%	18
	**2010**	7	88%	1	13%			0	0%	8
	**2011**	2	67%	1	33%			0	0%	3
	**2012**	5	100%	0	0%			0	0%	5
	**2013**	9	90%	0	0%			1	10%	10
	**2014**	9	90%	0	0%			1	10%	10
	**2015**	7	50%	0	0%			7	50%	14
	**2016**	25	56%	1	2%	16	36%	3	7%	45
	**2017**	12	25%	1	2%	29	60%	6	13%	48
	**2018**	11	27%	1	2%	26	63%	3	7%	41
	**2019**	13	24%	1	2%	31	56%	10	18%	55
	**2020**	9	15%	1	2%	38	64%	11	19%	59
	**2021**	6	9%	0	0%	52	81%	6	9%	64
	**2022**	13	21%	0	0%	37	61%	11	18%	61
	**2023**	20	35%	0	0%	35	61%	2	4%	57
	**2024**	9	18%	0	0%	29	58%	12	24%	50

[a] Inducible resistant DST procedure implemented in 2016 per recent CLSI recommendation.

		Susceptible		Intermediate		Resistant		Total
		N	Percent	N	Percent	N	Percent	N
**Ciprofloxacin**	**2008**	0	0%	5	50%	5	50%	10
	**2009**	8	44%	3	17%	7	39%	18
	**2010**	7	88%	1	13%	0	0%	8
	**2011**	2	67%	1	33%	0	0%	3
	**2012**	0	0%	1	20%	4	80%	5
	**2013**	2	20%	3	30%	5	50%	10
	**2014**	2	20%	3	30%	5	50%	10
	**2015**	1	7%	2	14%	11	79%	14
	**2016**	4	9%	10	22%	31	69%	45
	**2017**	6	13%	7	15%	35	73%	48
	**2018**	0	0%	0	0%	41	100%	41
	**2019**	0	0%	5	9%	50	91%	55
	**2020**	0	0%	1	2%	58	98%	59
	**2021**	2	3%	4	6%	58	91%	64
	**2022**	3	5%	9	15%	49	80%	61
	**2023**	0	0%	10	18%	47	82%	57
	**2024**	1	2%	1	2%	48	96%	50

		Susceptible		Intermediate		Resistant		Total
		N	Percent	N	Percent	N	Percent	N
**Amikacin**	**2008**	4	40%	6	60%	0	0%	10
	**2009**	7	39%	11	61%	0	0%	18
	**2010**	8	100%	0	0%	0	0%	8
	**2011**	3	100%	0	0%	0	0%	3
	**2012**	5	100%	0	0%	0	0%	5
	**2013**	10	100%	0	0%	0	0%	10
	**2014**	7	70%	0	0%	3	30%	10
	**2015**	7	50%	1	7%	6	43%	14
	**2016**	41	91%	2	4%	2	4%	45
	**2017**	48	100%	0	0%	0	0%	48
	**2018**	40	98%	1	2%	0	0%	41
	**2019**	54	98%	0	0%	1	2%	55
	**2020**	55	93%	3	5%	1	2%	59
	**2021**	62	97%	0	0%	2	3%	64
	**2022**	52	85%	1	2%	8	13%	61
	**2023**	56	98%	0	0%	1	2%	57
	**2024**	48	96%	1	2%	1	2%	50
**Doxycycline**	**2008**	0	0%	0	0%	10	100%	10
	**2009**	0	0%	0	0%	18	100%	18
	**2010**	2	25%	1	13%	5	63%	8
	**2011**	0	0%	0	0%	3	100%	3
	**2012**	0	0%	0	0%	5	100%	5
	**2013**	0	0%	0	0%	9	90%	10
	**2014**	0	0%	0	0%	10	100%	10
	**2015**	0	0%	0	0%	14	100%	14
	**2016**	4	9%	2	4%	39	87%	45
	**2017**	3	6%	3	6%	42	88%	48
	**2018**	0	0%	3	7%	38	93%	41
	**2019**	1	2%	3	5%	51	93%	55
	**2020**	0	0%	3	5%	56	95%	59
	**2021**	3	5%	4	6%	56	88%	64
	**2022**	2	3%	5	8%	54	89%	61
	**2023**	2	4%	3	5%	52	91%	57
	**2024**	3	6%	1	2%	46	92%	50

		Susceptible		Intermediate		Resistant		Total
		N	Percent	N	Percent	N	Percent	N
**Cefoxitin**	**2008**	–		–		–		nd
	**2009**	–		–		–		nd
	**2010**	–		–		–		nd
	**2011**	0	0%	1	33%	2	67%	3
	**2012**	1	20%	2	40%	2	40%	5
	**2013**	2	20%	6	60%	2	20%	10
	**2014**	2	20%	7	70%	1	10%	10
	**2015**	1	7%	10	71%	3	21%	14
	**2016**	7	16%	34	76%	4	9%	45
	**2017**	8	17%	26	54%	14	29%	48
	**2018**	2	5%	29	71%	10	24%	41
	**2019**	6	11%	42	76%	7	13%	55
	**2020**	2	3%	51	88%	5	9%	58
	**2021**	4	6%	53	84%	6	10%	63
	**2022**	11	18%	39	64%	11	18%	61
	**2023**	4	7%	44	77%	9	16%	57
	**2024**	1	2%	34	68%	15	30%	50

–, cefoxitin DST not performed.
